# CardioTwins: case report of a transcatheter mitral and tricuspid valve implantation in one patient

**DOI:** 10.1093/ehjcr/ytae336

**Published:** 2024-07-17

**Authors:** Tobias Schmidt, Karl-Heinz Kuck, Christoph Marquetand, Ingo Eitel, Christian Frerker

**Affiliations:** Department of Internal Medicine II, University Hospital of Schleswig-Holstein, Campus Lübeck, 23562 Lübeck, Germany; German Center for Cardiovascular Research (DZHK), Partner Site Hamburg–Kiel–Lübeck, Lübeck, Germany; Department of Internal Medicine II, University Hospital of Schleswig-Holstein, Campus Lübeck, 23562 Lübeck, Germany; Department of Internal Medicine II, University Hospital of Schleswig-Holstein, Campus Lübeck, 23562 Lübeck, Germany; German Center for Cardiovascular Research (DZHK), Partner Site Hamburg–Kiel–Lübeck, Lübeck, Germany; Department of Internal Medicine II, University Hospital of Schleswig-Holstein, Campus Lübeck, 23562 Lübeck, Germany; German Center for Cardiovascular Research (DZHK), Partner Site Hamburg–Kiel–Lübeck, Lübeck, Germany; Department of Internal Medicine II, University Hospital of Schleswig-Holstein, Campus Lübeck, 23562 Lübeck, Germany; German Center for Cardiovascular Research (DZHK), Partner Site Hamburg–Kiel–Lübeck, Lübeck, Germany

**Keywords:** Tricuspid valve, Mitral valve, Valve replacement, Case report

## Abstract

**Background:**

Besides transcatheter edge-to-edge repair (TEER), there are new interventional treatment options for mitral and tricuspid regurgitation in evaluation, such as a complete replacement of the valve through a prosthesis.

**Case summary:**

A 78-year-old previous coronary artery bypass graft-operated patient with symptomatic severe mitral regurgitation and tricuspid regurgitation was sequentially treated by a transfemoral transcatheter mitral and tricuspid valve prosthesis (Cardiovalve; Cardiovalve Ltd, Israel) due to unfavourable mitral valve anatomy. The transcatheter mitral valve implantation (TMVI) was performed first and after progression of the tricuspid regurgitation, a second transcatheter valve prosthesis was implanted in tricuspid position (TTVI) 1.5 years later. Imaging showed a twin look-alike picture of a mitral and tricuspid prosthesis and showing the possibility of a complete transcatheter based replacement of the mitral and tricuspid valve.

**Discussion:**

This case shows the possibility of a Cardiovalve prosthesis being used for TMVI and TTVI in a single patient. Especially in TEER ineligible patients, it might be a good treatment option after device approval.

**Learning points**

- To understand that intensive echocardiographic screening of patients with severe mitral regurgitation for evaluation of a mitral valve transcatheter edge-to-edge repair (M-TEER) procedure is important.
- Transcatheter mitral valve implantation could be an alternative treatment option for patients who are not eligible for M-TEER in the future.
- Transcatheter mitral valve implantation and interventional tricuspid valve implantation were feasible in a patient with a double valve disease.

## Introduction

**Figure 1:**
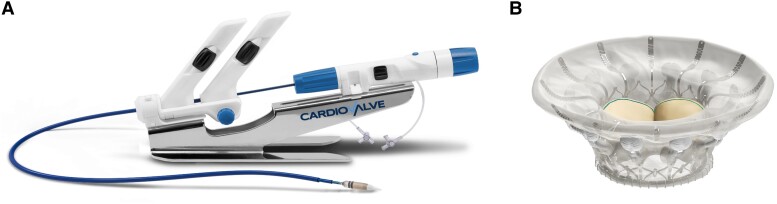
The Cardiovalve system. (*A*) Implantation catheter system; (*B*) Cardiovalve prosthesis.

Severe mitral or tricuspid regurgitation is known to be associated with an impaired survival.^1,2^ Transcatheter edge-to-edge repair (TEER) is nowadays a good therapeutic option for inoperable high-risk patients with severe mitral and/or tricuspid regurgitation. Nevertheless, there are unfavourable anatomies for a TEER procedure and therefore an interventional mitral valve implantation (TMVI) or an interventional tricuspid valve implantation (TTVI) might be a good option to achieve the best possible results.^3^ Instead of just repairing the mitral or tricuspid valve, there are more options coming up to completely replace the valve by an interventional transcatheter procedure.^4,5^ The Cardiovalve system (Cardiovalve Ltd, Israel) and prosthesis are designed to be delivered through a transfemoral approach and implanted under transoesophageal echocardiography and fluoroscopic guiding. It can be used for mitral as well as for tricuspid valve procedures (*Figure 1A* and *B*). In this case report, we present a TMVI and TTVI with an identical prosthesis in an ineligible mitral TEER patient.

## Summary figure

**Figure:**
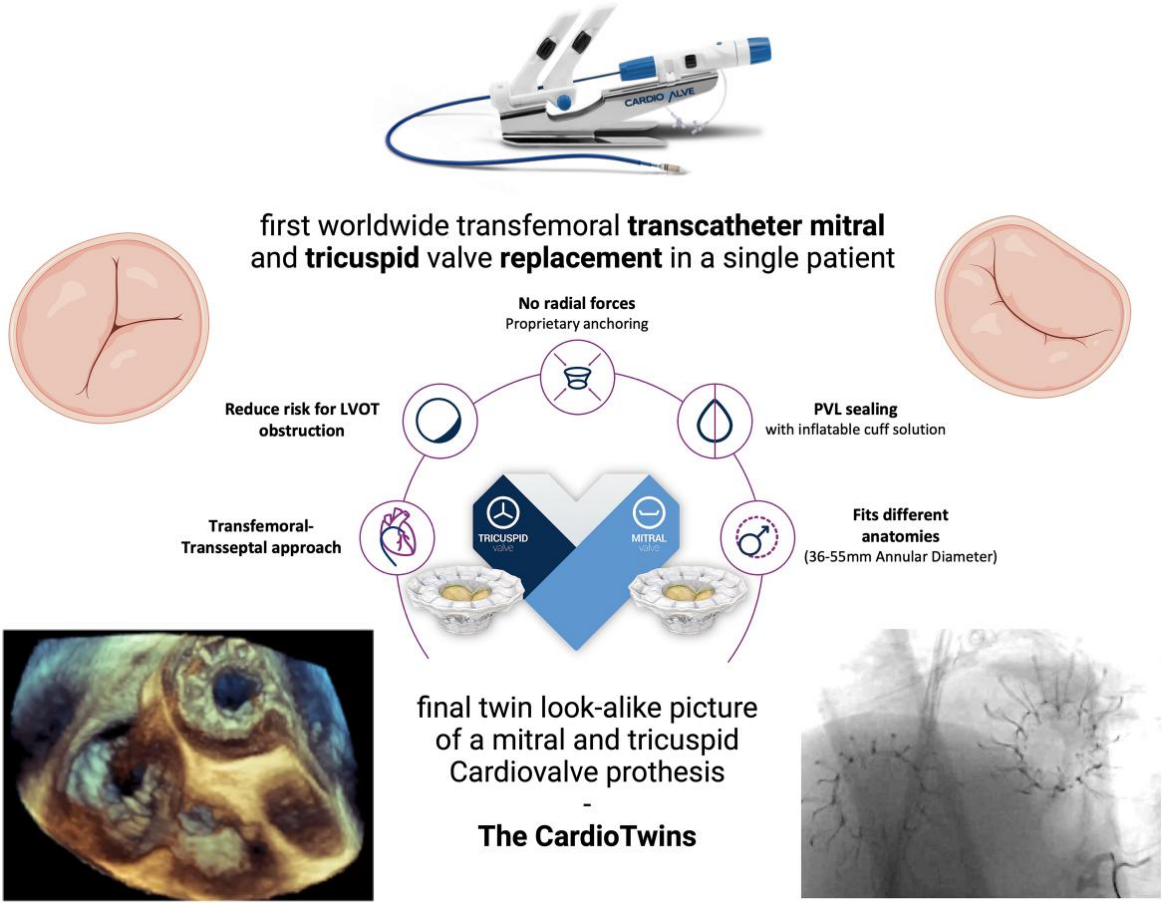


### Case presentation

**Figure 2:**
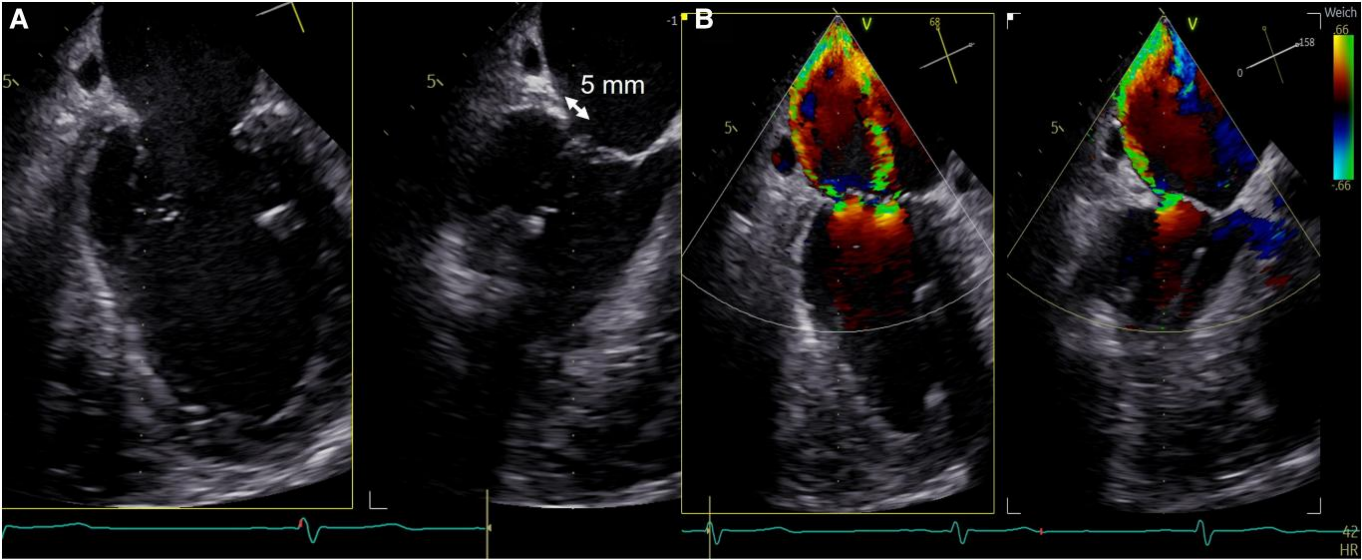
Baseline transoesophageal echocardiography, midesophageal commissural view of mitral valve. (*A*) Without colour Doppler, length of the posterior leaflet 5 mm; (*B*) severe eccentric mitral regurgitation. Before TMVI, there were no regional wall motion abnormalities of the LV with a left ventricular end-diastolic volume index of 84 mL/m².

**Figure 3:**
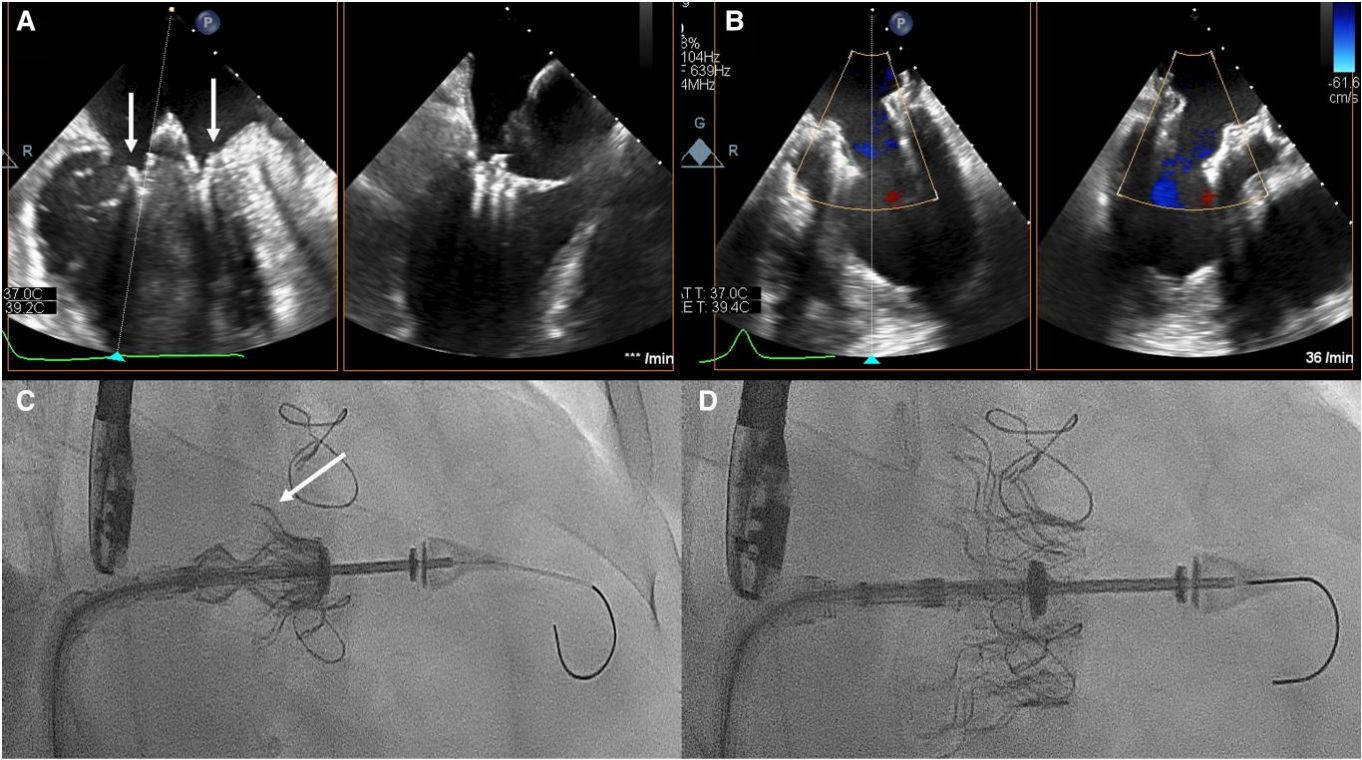
Implantation of the Cardiovalve prosthesis in mitral position. (*A*) Transoesophageal echocardiography in midesophageal commissural view of MV with opening of the ventricular legs (arrows); (*B*) transoesophageal echocardiography midesophageal commissural view of mitral valve final result without any mitral regurgitation; (*C*) fluoroscopy (projection RAO 26° and caudal 12°) showing open ventricular legs (arrow); (*D*) fluoroscopy (projection RAO 26° and caudal 12°) after final implantation.

A 78-year-old male patient with dyspnoea classified as New York Heart Association functional class III and peripheral oedema presented to our clinic with severe mitral regurgitation (MR) and moderate tricuspid regurgitation (TR). The patient underwent coronary artery bypass graft (CABG) surgery at the age of 55 years [left internal mammary artery—first marginal, right internal mammary artery–left anterior descending artery (LAD), vein graft—right coronary artery (RCA)] due to a severe coronary three-vessel disease with chronic total occlusion of the RCA, severe stenosis of the circumflex artery, and severe stenosis of the LAD with stenting of the left main in 2001 due to functional occluded bypasses to the LAD without any acute myocardial infarction. Furthermore, permanent atrial fibrillation, chronic renal disease stage III, type 2 diabetes mellitus, and a combined post- and pre-capillary pulmonary hypertension (mean pulmonary artery pressure 35 mmHg, pulmonary wedge pressure 24 mmHg, cardiac output 2.9 L/min, pulmonary vascular resistance 3.8 Wood units were derived from right heart catheterization) were documented. Coronary angiography excluded a progression of the coronary artery disease. The electrocardiogram showed previously known atrial fibrillation with regular ventricular response. Echocardiography confirmed a severe, extremely eccentric predominantly secondary MR [2D-effective regurgitation orifice area (EROA) 0.46 cm^2^, regurgitation volume 67 mL, vena contracta (VC) biplane 10 mm, mitral valve area of 4.8 cm^2^, mean pressure gradient was 1.5 mmHg] due to a dilation of the mitral valve annulus (intercommissural diameter was 36 mm) and an asymmetric tethering with a short posterior mitral leaflet of 5 mm length (*Figure 2A* and *B*) and a moderate-severe TR. Left ventricular ejection fraction was 52%, and N-terminal pro b-type natriuretic peptide (NT-proBNP) was elevated at 1901 ng/L. Before treatment, patient was on novel oral anticoagulant, betablocker, angiotensin receptor blocker, mineralocorticoid receptor antagonist, sodium-glucose transport 2-inhibitor, loop diuretics, and a distinct diabetic therapy. The EuroSCORE II was 10.4% and the STS score 16.7%. The interdisciplinary Heart Team decided for a TMVI procedure with a Cardiovalve prosthesis due to the fact of a high-risk patient for a re-operation (after prior CABG surgery), known pulmonary hypertension, and no need for a coronary bypass surgery. Mitral valve transcatheter edge-to-edge repair (M-TEER) was also not a good option due to the short and tethered posterior mitral valve leaflet. In addition, the possibility of a mitral valve replacement within the AHEAD study (NCT03339115; European Feasibility Study of the Cardiovalve Transfemoral Mitral Valve System) was another fact for the decision of treatment. The anchoring mechanism is a clamping of the valve leaflets with ventricular legs and an atrial flange to anchor from the atrial side (see also *Figure 1A* and *B*). A computer tomography (CT) scan was done for sizing of the mitral valve annulus. CT evaluation included also the neo-left ventricular outflow tract (LVOT) narrowing and the access venous vessel. After approval of the independent clinical screening committee and patient informed consent, a Cardiovalve prosthesis (size M) was implanted via a transfemoral–transseptal access. Surgical cut-down was recommended in the first cases regarding safety issues due to the 37 Fr sheath size. After performing a transseptal puncture as posterior and superior as possible, a pre-dilation of the septum with a 10 × 40 mm peripheral transluminal angioplasty balloon (Admiral; Medtronic, Germany) was done. Thereafter, a steerable sheath (8 Fr) and a pigtail catheter were used for crossing of the mitral valve. The pigtail catheter was exchanged for a stiff wire (Lunderquist; Cook Medical, Ireland). Over this wire, a floating manoeuvre using a Reliant balloon (Medtronic, Germany) to ensure a free positioning without any interaction with the cords was performed. Thereafter, the delivery system was advanced into the left atrium and centred with respect to the mitral annulus, the ventricular grasping legs were opened, dived into the left ventricle, and afterwards retracted (*Figure 3A*). After confirmation that all ventricular legs grasped the leaflets, the atrial flange was opened followed by releasing of the ventricular part of the system (*Figure 3* and Supplementary material online, *Video S1*). Echocardiography showed a perfect result with only trace paravalvular MR and a mean gradient of 4.5 mmHg. There was no relevant LVOT gradient after the procedure (mean LVOT 1.6 mmHg). The right heart catheter measurement revealed no relevant left-to-right atrial shunt (Qp:Qs = 1.1), and the shunt was kept open. Patient was extubated right after the procedure and discharged to a rehab clinic on Day 11. For prevention of a thrombus, oral anticoagulation with warfarin was administered with an international normalized ratio (INR) of 2.5–3.5.

**Figure 4:**
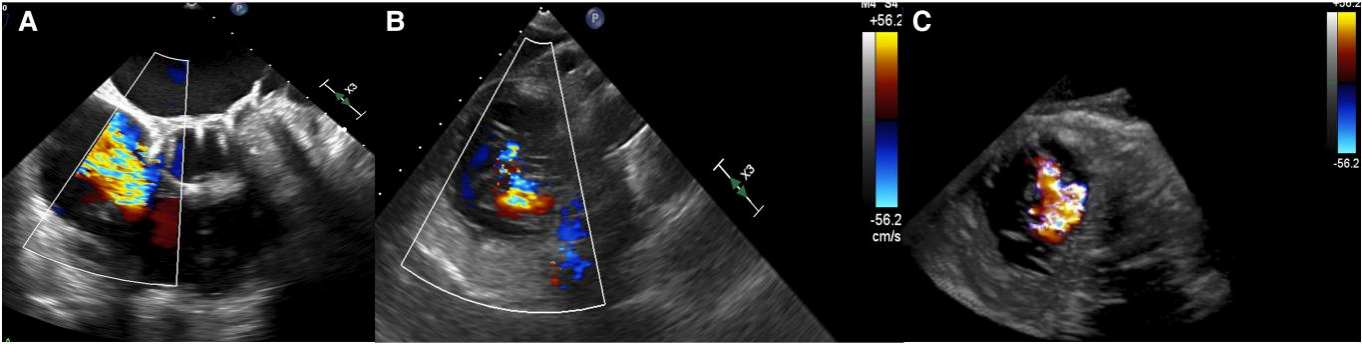
Transoesophageal echocardiography after 1.5 years showing severe tricuspid regurgitation. (*A*) Right ventricle inflow-outflow view; (*B*) transgastric view; (*C*) 3D view. Before TTVI, basal right ventricular diameter was 53 mm with a longitudinal diameter of 76 mm. RV function had a TAPSE of 14 mm with a RV-S′ of 11 cm/s and a RV-FAC of 37.5%.

**Figure 5:**
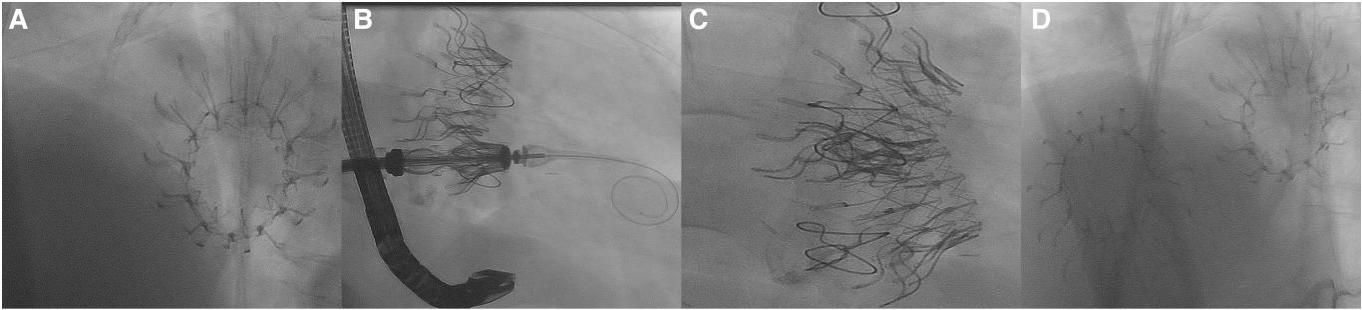
Fluoroscopy of implantation of the Cardiovalve prosthesis in tricuspid position. (*A*) At baseline with only the Cardiovalve in mitral position (projection LAO 70°); (*B*) opening of the ventricular legs of the tricuspid Cardiovalve system (projection RAO 24°); (*C*) CardioTwins with Cardiovalve sitting in mitral and triscuspid position long axis view (projection RAO 20°); (*D*) CardioTwins with Cardiovalve sitting in mitral and triscuspid position short axis view (projection LAO 70°).

During the follow-up period, the patient’s symptoms regarding dyspnoea improved but peripheral oedema was still present. A new echocardiography 1.5 year later showed a progression of the secondary TR due to an annulus dilation (septal–lateral diameter 54 mm and anterior–posterior diameter was 40 mm; VC biplane 10 mm, PISA–EROA 0.44 cm^2^, reg. volume 48 mL) (*Figure 4A–C*) with good leaflet lengths (septal leaflet 18 mm, anterior leaflet 22 mm, and posterior leaflet 24 mm). Estimated pulmonary artery systolic pressure was 43 mmHg (plus central venous pressure). The mitral prosthesis still showed no paravalvular leakage (PVL) and a gradient of 3.5 mmHg. The case was again discussed within our heart team and after successful replacement in mitral position, the patient wanted to be screened for a Cardiovalve implantation in tricuspid position within the TARGET trial (NCT05486832; Safety and Performance of the Cardiovalve TR Replacement System) even though there were no complicating facts against a TEER procedure for the tricuspid valve. On the basis of a positive result for the possibility of an interventional replacement of the tricuspid valve in the TARGET study, implantation was performed after careful evaluation including discussion in the screening committee of the study. The implantation steps were similar to the mitral side despite avoiding of transseptal puncture and using of a Safari wire (Boston Scientific; Germany) instead of a Lunderquist. The patient received a Cardiovalve L prosthesis (*Figure 5A–D* and Supplementary material online, *Video S1*). Echocardiographic evaluation directly after the implantation showed again a perfect result with only a trace paravalvular leakage and a gradient of 1 mmHg. The patient was discharged home after 8 days, again on warfarin with an INR of 2.5–3.5. At nine months of follow-up after the second Cardiovalve implantation, echocardiographic images showed no PVL for both prosthesis and a gradient of 4 mmHg over the mitral and a gradient of 2–3 mmHg over the tricuspid prosthesis.

## Discussion

Today, MR is the most prevalent valve disease in the Western world.^2^ The current ESC/EACTS and the AHA/ACC guidelines recommend a M-TEER procedure in high-risk patients with symptomatic severe MR.^6,7^ However, not every patient is eligible for such a procedure due to anatomical reasons. There are different screening selection criteria for a M-TEER procedure and unsuitable anatomies like leaflet clefts, significant leaflet tethering, leaflet configuration, or leaflet-to-annulus index known.^6^ One important criterium is the length of the leaflets. A leaflet length of >10 mm is optimal in comparison to a length of <7 mm that should be avoided or limited for extremely high experienced centres. There are only limited data existing so far for long-term durability and re-interventions after TEER procedures. In a scenario where anatomic features of the native valve are not ideal for TEER, replacement could be a more effective treatment option. Furthermore, re-intervention after a TEER procedure could be difficult.

There are already some data from the CHOICE MI study that TMVI is a reasonable alternative, especially in patients being ineligible for M-TEER.^3^ Transcatheter mitral valve implantation showed good MR elimination and sustained functional improvement at 1 year.^8,9^ Until today, only one TMVI system is commercially available (Tendyne™; Abbott Vascular, Germany). However, this prosthesis requires transapical access. There are different types of transcatheter prosthesis that can be implanted via a transfemoral–transseptal approach currently within early feasibility studies available. One of these systems is the Cardiovalve prosthesis, which can be implanted in mitral but also in tricuspid position. The prosthesis consists of two elements for the anchoring mechanism; the ventricular legs are necessary to achieve the attachment with the leaflets from the ventricular side and an atrial flange to cover and anchor from the atrial side. In the middle of the device, the three-scallop-shaped bovine pericardial leaflets are sutured to function as a complete replaced valve. The two elements create 24 grasping points that fixate the device to the native tricuspid/mitral annulus. The prosthesis is available in three sizes from 35–45 mm (size M) to 42–50 mm (size L) up to 47–55 mm (size XL; in mitral up to 53 mm) (see also *Figure 1A* and *B*). These devices are only available in early feasibility studies, therefore screening and implantation can only be performed within the study. Annulus, ventricular and left ventricular outflow tract sizes, severe annulus or leaflet calcification, and the need for oral anticoagulation as well as echocardiographic windows for procedural imaging in addition to the inclusion criteria of the studies are limiting factors today. Oral anticoagulation is especially in elderly patients an important issue that must to be discussed before the procedure, since until today the need of oral anticoagulation is given for these patients, while direct oral anticoagulants are possible. This is different to TEER procedures in which antiplatelet therapy is possible (on a low level of evidence).

In our patient with an ineligible anatomy for a M-TEER procedure due to the short posterior leaflet length, we had the option to treat the MR with TMVI within the AHEAD study.

Due to the satisfaction of our patient after treatment of the mitral valve with Cardiovalve prosthesis, we screened the patient for transcatheter tricuspid valve implantation (TTVI). For tricuspid regurgitation, the EVOQUE (Edwards Lifesciences, Irvine, CA) prosthesis just became commercially available and the TRISCEND study showing low mortality rates for the procedure and during follow-up to one-year with low rates of hospitalization especially in an older patient population.^5^ The TARGET trial evaluates the safety and feasibility of the Cardiovalve prosthesis in patients with severe TR. At two months of follow-up after the second Cardiovalve implantation, echocardiographic images showed no PVL for both prosthesis and a gradient of 5 mmHg above the mitral and a gradient of 3 mmHg above the tricuspid prosthesis.

In summary, we report on the first patient worldwide, who received serial TMVI and TTVI using the Cardiovalve prosthesis with finally showing a Twin look-alike picture and good functional results.

## Supplementary Material

ytae336_Supplementary_Data

## Data Availability

The data underlying this article are available in the article and in its online Supplementary material.

## Lead author biography

T. Schmidt is an interventional cardiologist specialized in transcatheter heart valve interventions.

## Supplementary material

**Consent**: Patient consented to an anonymized publication according to the COPE guidelines.

**Funding:** No funding.
